# Measurement and Evaluation of Finger Tapping Movements Using Log-linearized Gaussian Mixture Networks

**DOI:** 10.3390/s90302187

**Published:** 2009-03-26

**Authors:** Keisuke Shima, Toshio Tsuji, Akihiko Kandori, Masaru Yokoe, Saburo Sakoda

**Affiliations:** 1 Graduate School of Engineering, Hiroshima University, 1-4-1 Kagamiyama, Higashi-Hiroshima, Hiroshima, Japan; E-Mail: tsuji@bsys.hiroshima-u.ac.jp; 2 Advanced Research Laboratory, Hitachi Ltd., 2520 Hatoyama, Saitama, Japan; E-Mail: akihiko.kandori.vc@hitachi.com; 3 Graduate School of Medicine, Osaka University, 1-1 Yamadaoka, Suita, Osaka, Japan; E-Mails: myokoe@neurol.med.osaka-u.ac.jp(M.Y.); sakoda@neurol.med.osaka-u.ac.jp(S.S.)

**Keywords:** Finger tapping movements, magnetic sensors, neural networks, pattern discrimination, diagnosis support

## Abstract

This paper proposes a method to quantitatively measure and evaluate finger tapping movements for the assessment of motor function using log-linearized Gaussian mixture networks (LLGMNs). First, finger tapping movements are measured using magnetic sensors, and eleven indices are computed for evaluation. After standardizing these indices based on those of normal subjects, they are input to LLGMNs to assess motor function. Then, motor ability is probabilistically discriminated to determine whether it is normal or not using a classifier combined with the output of multiple LLGMNs based on bagging and entropy. This paper reports on evaluation and discrimination experiments performed on finger tapping movements in 33 Parkinson’s disease (PD) patients and 32 normal elderly subjects. The results showed that the patients could be classified correctly in terms of their impairment status with a high degree of accuracy (average rate: 93.1 ± 3.69%) using 12 LLGMNs, which was about 5% higher than the results obtained using a single LLGMN.

## 1. Introduction

Assessment of neurological disorders such as Parkinson’s disease (PD) symptoms through blood tests or clinical imaging procedures (including computed tomography scanning and magnetic resonance imaging) cannot fully determine the severity of the disease [1]. However, traditional symptom evaluations made from doctor’s inquiries into the patient’s status, or complaints from patients themselves, are unable to quantitatively assess disease symptom development, and potentially overlook significant changes in the patient’s condition. Quantitative evidence is thus required for diagnosis support and clinical assessment by evaluating clinical semiology and drug therapy efficacy.

To identify neurological disorders such as PD and quantify motor function by sensing patients’ physical movements, various assessment methods have been discussed, including tremor and reaction movements [2], repetitive eye-hand movements [3] and finger tapping movements [4]. In particular, finger tapping movements have already been widely investigated, and a method to analyze tapping rhythm as well as a method to quantify tapping amplification and velocity [1, 5–8] have been reported. The above evaluations, however, were performed only for basic analysis such as verification of the feature quantities of PD patients. To realize a method of measurement and evaluation for use in the routine assessment of PD in clinical environments, the features of finger movements need to be ascertained numerically for quantitative classification and evaluation.

The classification and evaluation of a patient’s symptom severity are reduced to a clustering problem involving measured data distribution. So far, several nonlinear classification methods have been proposed by assuming probabilistic distribution of measured data, and probabilistic neural networks (PNNs) have recently attracted widespread attention [9, 10]. In particular, the log-linearized Gaussian mixture network (LLGMN) [10] proposed by Tsuji *et al.* has been widely utilized for pattern classification problems of various bioelectric signals. However, no reports have been published concerning the use of such PNNs to classify motility disturbance.

On the other hand, the movements of PD sufferers are highly influenced by differences between the symptoms of individual patients. Furthermore, similar movements cannot be observed in the same patient group, and the relationships between the features of movement and the severity of the disease have not been clearly identified from previous studies. It may therefore be difficult to classify the disease and symptoms using a single PNN from the features of movement extracted. To overcome these problems, a system is required that meets the following criteria: (i) the user’s movements can be quantitatively assessed to detect the severity of the disease and the effectiveness of medication; (ii) each movement feature can be individually evaluated and visually confirmed; and (iii) the symptoms and disease of the subject can be comprehensively discriminated by combing the evaluation results and features.

The purpose of this study is to realize a system to support motor function diagnosis; to this end, we propose a novel method to measure and evaluate finger tapping movements and PD by employing multiple LLGMNs. The system utilizes a magnetic sensor developed by Kandori *et al.* [8] to measure finger tapping movements and extract its features (such as velocity and rhythm), and then displays the feature indices for doctors’ reference on a monitor. Further, the relationships between symptoms and movement features are embedded into the neural networks through learning, and can be used to evaluate motor function by combining outputs of multiple neural networks based on the ensemble learning method of bagging [11]. Since the outcomes of the neural networks indicate probabilities, doctors can intuitively understand the features of finger tapping movements and make quantitative evaluation from the indices or radar chart on the display.

In this paper, the structure and algorithm of the proposed measurement and evaluation method are explained in Section 2. Section 3 describes the experiments conducted to show the effectiveness of the method, and finally, Section 4 concludes the paper.

## 2. Diagnosis support system for finger tapping movements

**Figure 1.**
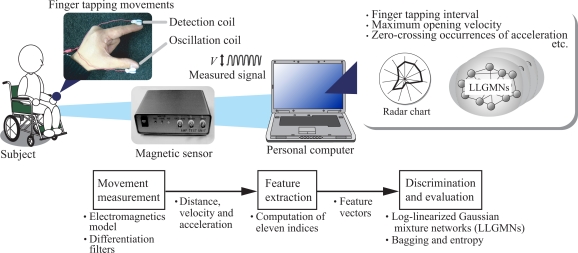
Concept of the proposed diagnosis support system for finger tapping movements.

Figure 1 shows the proposed diagnosis support system, which consists of a magnetic sensor and a personal computer. The subject conducts finger tapping movements with two magnetic sensor coils attached to his or her fingers, and the system measures the distance between the two fingertips (the *fingertip distance*). The features and evaluation indices of the measured movements are computed from the fingertip distances, and are then discriminated and evaluated using multiple LLGMNs after standardization based on normal subject movements. All features, evaluation indices and discrimination results are displayed on a monitor, enabling the doctor to assess motor function through finger tapping tests. The details of each process are explained in the following subsections.

### 2.1. Movement measurement

**Figure 2.**
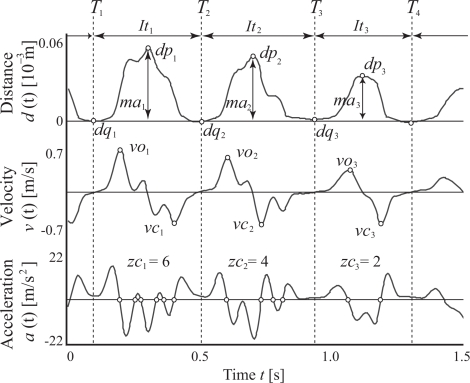
Examples of the measured signals. [12]

The magnetic sensor developed by Kandori *et al*. [8] is utilized to measure finger tapping movements. This sensor can output a voltage corresponding to changes in distance between the detection coil and the oscillation coil by means of electromagnetic induction. First, the two coils are attached to the distal parts of the user’s fingers, and finger tapping movements are measured. The fingertip distances are then obtained from the output voltage by a calibration model expressed as [12]

(1) $$d(t)=\alpha \tilde{V}(t)-\varepsilon$$

(2) $$\tilde{V}(t)=V^{-\frac{1}{3}}(t),$$

where *d*(*t*) denotes the fingertip distance, *V* (*t*) is the measured voltage of the sensors at a given time *t*, *Ṽ* (*t*) is the reciprocal number for the cubic root of *V* (*t*) for linearization, and *α* and *ε* are constants computed from calibration. In the calibration process, *α* and *ε* are estimated using the linear least-square method for *n* values of measured output voltages and fingertip distances for each subject. The calibration process can reduce the influence of the slope of the coils and modeling errors. Further, the velocity *v*(*t*) and acceleration *a*(*t*) can be calculated from the fingertip distance *d*(*t*) using differentiation filters (see Figure 2 [12]).

### 2.2. Feature extraction

This paper defines eleven indices for the evaluation of finger tapping movements as follows:

1. Total tapping distance
2. Average maximum amplitude of finger taps
3. Coefficient of variation (CV) of maximum amplitude
4. Average finger tapping interval
5. CV of finger tapping interval
6. Average maximum opening velocity
7. CV of maximum opening velocity
8. Average maximum closing velocity
9. CV of maximum closing velocity
10. Average zero-crossing number of acceleration
11. Spectral variability of finger taps

The integration of the absolute value of velocity *v*(*t*) throughout the measurement time is signified as the total tapping distance (Index 1). The contact time between fingers is also determined from *d*(*t*), *v*(*t*) and *a*(*t*). First, the threshold *M^th^* is calculated as

(3) $$\begin{array}{c} M^{\mathrm{th}}=\{\begin{array}{ll} {\tilde{M}}^{\mathrm{th}} & \left({\tilde{M}}^{\mathrm{th}}\geq \zeta \right)\\ \zeta & \left({\tilde{M}}^{\mathrm{th}}<\zeta \right) \end{array}\\ {\tilde{M}}^{\mathrm{th}}=\eta (\frac{1}{K}\sum_{k=1}^{K}d_{k}^{\max}-\frac{1}{J}\sum_{j=1}^{J}d_{j}^{\min}) \end{array}$$

where *ζ* and *η* are constants determined by the minimum and maximum values of all subjects’ fingertip distances, respectively, $d_{k}^{\max}$ denotes the distance between fingertips at the *k*th time when *v*(*t*) = 0 and *a*(*t*) < 0 in the measurement time window, and $d_{j}^{\min}$ denotes the same at the *j*th time when *v*(*t*) = 0 and *a*(*t*) > 0; and *K* and *J* are the number of $d_{k}^{\max}$ and $d_{j}^{\min}$, respectively. Then, the *i*th time at which the distance $d_{j}^{\min}$ falls below the threshold *M^th^* is defined as the contact time *T_i_* (*i* = 1, 2,. . ., *I*, where *I* is the number of contacts between fingertips).

As feature quantities of the *i^th^* tapping, the finger tapping interval *It_i_* (i.e., the time interval between two consecutive contacts) is defined as *It_i_* = *T_i_*_+1_ *− T_i_*. The maximum and minimum amplitude points (*dp_i_*, *dq_i_*) between the time interval [*T_i_*, *T_i_*_+1_] are calculated from the measured fingertip distance *d*(*t*), and the average (Index 2) and CV (Index 3) of maximum amplitudes *ma_i_* = *dp_i_* *− dq_i_* are computed for the measurement time. Further, the positive and negative maximum velocities between the time interval [*T_i_*, *T_i_*_+1_] are defined as the maximum opening velocity *vo_i_* and the maximum closing velocity *vc_i_*, respectively. The averages and CVs of the finger tapping interval, maximum opening velocity and maximum closing velocity are then computed from all the values of *It_i_*, *vo_i_*, and *vc_i_* (Indices 4–9), respectively.

**Figure 3.**
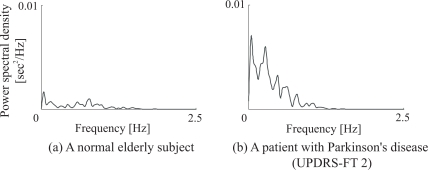
An example of the spectral variability of finger taps, note that UPDRS-FT 2 stands for the Unified Parkinson’s Disease Rating Scale part III finger tapping score 2. [13]

In addition, *zc_i_*, which denotes the number of zero crossings of the acceleration signal *a*(*t*), is calculated from each time interval between *T_i_* and *T_i_*_+1_, and the number of zero-crossing points of acceleration *zc_i_* are defined as the evaluation values of multimodal movements (Index 10). Finally, the spectra of the finger tapping intervals are calculated by applying heart rate variability analysis to evaluate the variability of finger tapping rhythms. The time-series finger tapping interval *It_i_* is resampled at *f_a_* Hz by applying the linear interpolation method, and the power spectral density of the data is then estimated using the fast Fourier transform (FFT). Figure 3 shows an example of spectral variability in finger taps. Figures 3(a) and (b) describe the analysis results for a normal elderly subject and a PD patient (UPDRS-FT 2: Unified Parkinson’s Disease Rating Scale [13] part III finger tapping score 2), respectively. UPDRS-FT is a way of evaluating Parkinson’s symptoms, and is determined based on visual inspection by a doctor. UPDRS-FT scores are defined as follows:

(0): Normal;
(1): Mild slowing and/or reduction in amplitude;
(2): Moderately impaired. Definite and early fatiguing. May have occasional arrests in movement;
(3): Severely impaired. Frequent hesitation in initiating movements or arrests in ongoing movement;
(4): Can barely perform the task.

It can be seen that the power spectrum of the PD patient is larger than that of the normal elderly subject. Therefore, the value of the integrated power spectrum from *f_b_* to *f_c_* Hz is defined as the spectral variability of finger taps (Index (11)), where *f_b_* and *f_c_* are constants determined by the spectra of all subjects’ finger taps.

The evaluation indices calculated for the subject are normalized based on the indices of normal subjects to enable comparison of the differences in movements. Since it was observed from the preliminary experiment that three evaluation indices of PD patients (i.e., average maximum amplitude, maximum opening velocity and maximum closing velocity) were smaller than those of normal elderly subjects, these indices were used to calculate the inverse number for every single tap, and the total tapping distance was converted to its inverse number. Calculating the inverse number for the above indices, all the indices of PD patients thus become larger than those of normal elderly subjects. Then, each index is converted to the standard normally distributed variable *x_j_* using the mean and standard deviations of the index values measured from the normal subjects:

(4) $$x_{j}=(z_{j}-\mu_{j})/\sigma_{j}.$$

Here, *j* corresponds to the index number, *z_j_* is the computed value in each index, and *μ_j_* and *σ_j_* describe the average and standard deviations of each index in the group of normal elderly subjects respectively. *j* = 1 represents the total tapping distance, *j* = 2, . . ., 9 signify the averages and CVs of maximum amplitude, finger tapping interval, maximum opening velocity and maximum closing velocity, and *j* = 10 and 11 denote the average zero-crossing number of acceleration and the spectral variability of finger taps. Additionally, the vector ***x*** = [*x*_1_, *x*_2_, . . ., *x*_11_]^T^ is defined for the discrimination of finger tapping movements.

### 2.3. Evaluation using probabilistic neural network ensembles

The extracted features are discriminated to enable evaluation of motor function. In this paper, a log-linearized Gaussian mixture network (LLGMN) [10] proposed by Tsuji *et al*. is used as the PNN, and each index is evaluated using the ensemble learning method based on bagging [11] for the LLGMN. In the following subsection, the details of the LLGMN structure and the combination method for LLGMNs are explained.

#### 2.3.2. LLGMN [10]

The LLGMN is based on the Gaussian mixture model (GMM) and the log-linear model of the probability density function (pdf), and the *a posteriori probability* is estimated based on the GMM by learning. By applying the log-linear model to a product of the mixture coefficient and the mixture component of the GMM, a semiparametric model of the pdf is incorporated into a three-layer feed-forward PNN. Through learning, the LLGMN distinguishes movement patterns with individual differences, thereby enabling precise pattern recognition for bioelectric signals such as EMG and EEG [10, 14, 15].

**Figure 4.**
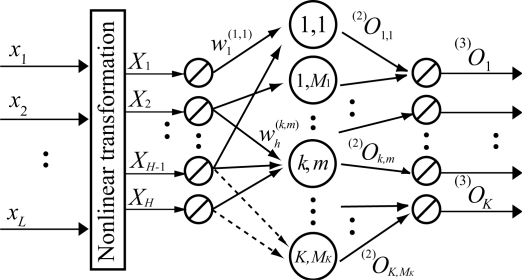
Structure of the LLGMN. [10]

The structure of the LLGMN is shown in Figure 4. First, the input vector ***x*** ∈ ℜ*^d^* is converted into a modified vector ***X*** ∈ ℜ*^H^* as follows:

(5) $$X={\left[1,x^{T},x_{1}^{2},x_{1}x_{2},\cdots ,x_{1}x_{d},x_{2}^{2},x_{2}x_{3},\cdots ,x_{2}x_{d},\cdots ,x_{d}^{2}\right]}^{T}$$

where *x_i_*, *i* = 1, 2, . . ., *d*, are the elements of ***x*** and *H* = 1 + *d*(*d* + 3)/2. The first layer consists of *H* units corresponding to the dimension of ***X***, and the identity function is used for the activation of each unit. The relationship between the input ^(1)^*I_h_* and the output ^(1)^*O_h_* of each unit in the first layer is defined as

(6) $${}^{\left(1\right)}O_{h}{=}^{\left(1\right)}I_{h}=X_{h}.$$

In the second layer, each unit receives the output of the first layer weighted by the weight $w_{h}^{\left(k,m\right)}$ (*h* = 1, 2, . . ., *H*; *k* = 1, . . ., *K*; *m* = 1, . . ., *M_k_*) and outputs the *a posteriori* probability of each Gaussian component. Here, *K* denotes the number of classes, and *M_k_* is the number of Gaussian components in class *k*. The relationships between the input of unit {*k*, *m*} in the second layer ^(2)^*I_k,m_* and the output ^(2)^*O_k,m_* are defined as

(7) $${}^{\left(2\right)}I_{k,m}=\sum_{h=1}^{H}{}^{\left(1\right)}O_{h}w_{h}^{\left(k,m\right)}$$

(8) $${}^{\left(2\right)}O_{k,m}=\frac{\text{exp}{[}^{\left(2\right)}I_{k,m}]}{\sum_{k^{\prime }=1}^{K}\sum_{m^{\prime }=1}^{M_{k^{\prime }}}\text{exp}{[}^{\left(2\right)}I_{k^{\prime },m^{\prime }}]},$$

where $w_{h}^{\left(K,M_{k}\right)}=0\;(h=1,\ldots ,H)$.

The third layer consists of *K* units corresponding to the number of classes. The unit *k* sums up the outputs of *M_k_* components {*k*, *m*} in the second layer. The function between the input and the output is described as

(9) $${}^{\left(3\right)}O_{k}{=}^{\left(3\right)}I_{k}=\sum_{m=1}^{M_{k}}{}^{\left(2\right)}O_{k,m},$$

where the output ^(3)^*O_k_* corresponds to the *a posteriori* probability of class *k*.

#### 2.3.2. Combination rules of LLGMNs

**Figure 5.**
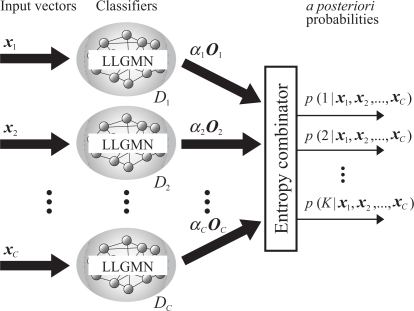
Strategy for combining LLGMNs

The combination strategy for multiple LLGMNs is shown in Figure 5. This method consists of the *C* LLGMN classifiers, corresponding to the number of input vector *x_c_*. Each LLGMN outputs the *a posteriori* probability of each learned class, which are then weighted and combined based on the ensemble learning method of bagging [11] and entropy. The networks can evaluate the degree of influence and the effect of each input vector for the classification because the entropy describes the ambiguity of each LLGMN output.

First, each input vector ***x_c_***(*c* = 1, 2, . . ., *C*) is input to the *c*th LLGMN, and the *a posteriori* probability vector ***O_c_*** is calculated by the LLGMN. Here, ***O_c_*** is defined by Equation 10 using the *a posteriori* probability *p*(*k|****x_c_***) at given value ***x_c_***:

(10) $$O_{c}={{[}^{\left(3\right)}O_{1}{,}^{\left(3\right)}O_{2},\ldots^{\left(3\right)}O_{K}]}^{T}={\left[p(1|x_{c}),\;p(2|x_{c}),\ldots ,p(K|x_{c})\right]}^{T}.$$

The entropy combinator receives the output of each LLGMN weighted by coefficient *α_c_*, and outputs the *a posteriori* probabilities of all classes. Each element of the entropy combinator’s input vector ***y_c_*** is given by

(11) $$y_{k}(x_{c})=\alpha_{c}\;p(k|x_{c}),$$

where coefficient *α_c_* (0 < *α_c_* < 1), which denotes the degree of effect of the *c*th LLGMN’s output, is defined as

(12) $$\alpha_{c}=1-H(x_{c})=1+\frac{1}{{\text{log}}_{2}K}\left(\sum_{k=1}^{K}p(k|x_{c})\;{\text{log}}_{2}\;p(k|x_{c})\right).$$

Here, *H*(***x_c_***) signifies the entropy of the output of the LLGMN, and denotes the ambiguity of the *a posteriori* probabilities. When these probabilities are ambiguous, the entropy *H*(***x_c_***) becomes large and *α_c_* approaches 0.

In the entropy combinator, the *a posteriori* probabilities of all classes are calculated by

(13) $$Y_{k}=p(k|x_{1},x_{2},\ldots ,x_{C})=\frac{\sum_{c^{\prime }=1}^{C}y_{k}({x^{\prime }}_{c})}{\sum_{k^{\prime }}^{K}\sum_{c^{\prime }=1}^{C}y_{k}({x^{\prime }}_{c})}.$$

In the above method, each pdf for input vector ***x_c_*** can be estimated and combined, and the networks can be used to calculate the *a posteriori* probability of class *k* for any given measured data. Further, since *α_c_* is equivalent to the degree of influence of each input vector for classification, each input vector can be evaluated using *y_k_*(***x_c_***).

For the discrimination of measured data, the entropy of all classes defined by Eq. 14 is used:

(14) $$E=-\sum_{k=1}^{K}Y_{k}\;{\text{log}}_{2}\;Y_{k}.$$

If *E* is smaller than discrimination determination threshold value *E_d_*, the class with the highest *a posteriori* probability becomes the result of discrimination. Otherwise, if *E* exceeds *E_d_*, discrimination is suspended as an obscure class. *E_d_* is a constant determined by trial and error.

#### 2.3.2. Evaluation of finger tapping movements

The finger tapping movements conducted by the user are evaluated and classified using the above neural networks. First, input vector ***x_c_*** is created from measured finger tapping movements for their evaluation. ***x***(*t_all_*) ∈ ℜ^11^ and ***x***(*t_d_*) ∈ ℜ^11^, which are the feature vectors, are computed for the overall measurement time *t_all_* and the time interval [ $t_{d}^{\mathrm{st}}$, $t_{d}^{\mathrm{ed}}$] (*d* = 1, 2, . . . ., *D*) respectively. Then, the *j*th elements *x_j_*(*t_d_*) of ***x***(*t_d_*) (*d* = 1, 2, . . . ., *D*) are used to make the new vector, defined as ***x*′***_j_* = [*x_j_*(*t*_1_), *x_j_*(*t*_2_), . . ., *x_j_*(*t_D_*)]^T^ ∈ ℜ*^D^*(*j* = 1, 2, . . ., 11). Here, $t_{d}^{\mathrm{st}}$, $t_{d}^{\mathrm{ed}}$, and *D* are constants determined by all subjects’ finger tapping movements to enable evaluation of movement behavior according to fatigue in subjects.

The system next measures the finger tapping movements of the patient and those of normal subjects. The feature vectors ***x*′***_j_* and ***x***(*t_all_*) calculated from these movements are then input to each LLGMN as teacher vectors, and the LLGMNs are trained to estimate the *a posteriori* probabilities of each movement. Thus, the number of LLGMNs is *C* = 11 + 1 = 12. After training, the system can calculate similarities between patterns in the subject’s movements and trained movements as *a posteriori* probabilities by inputting the newly measured vectors to the LLGMNs. The significance of each feature (i.e., the input vector) on motor function could also be evaluated by computing the degree of influence *α_c_* for each classifier.

## 3. Experiments

**Figure 6.**
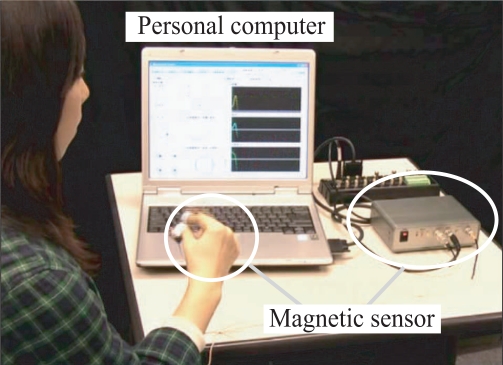
The prototype system developed and the experimental setup.

To verify the effectiveness of the proposed system, we developed a prototype and performed discrimination experiments on the measured finger movements. Figure 6 shows the prototype developed and the setup of the experiment conducted using it.

### 3.1. Methods

The subjects were 33 patients with PD (male: 16, female: 17, average age: 69.4 ± 8.1 years) and 32 normal elderly subjects (male: 16, female: 16, average age: 68.2 ± 5.0 years). The subjects were directed to assume a sitting posture at rest. Coils were attached to the distal parts of the thumb and index finger, and the magnetic sensor was calibrated using three calibration values of 20, 30 and 90 mm or 20, 30 and 60 mm. After a brief finger tapping movement trial using both the left and right hands, the movement of each hand was measured for 60 s in compliance with instructions to move the fingers as far apart and as quickly as possible. The severities of PD in the patients were evaluated by a neurophysician based on UPDRS-FT [13] (see *2.2. Feature extraction*). The investigation was approved by the local Ethics Committee, and informed consent was obtained from all subjects. The calculated indices were standardized on the basis of the values obtained from the normal elderly subjects. The parameters used in the analysis were *η* = 0.1, *ζ* = 5 mm, *f_a_* = 10 Hz, *f_b_* = 0.2 Hz, *f_c_* = 2.0 Hz and the sampling frequency was 100 Hz.

Each index was computed for the overall measurement time *t_all_* = 60 s and at four pre-specified time intervals of *t*_1_ = [0, 30], *t*_2_ = [10, 40], *t*_3_ = [20, 50] and *t*_4_ = [30, 60] and input to the LLGMNs. The measured finger tapping movements were then put into two classes in terms of whether they were normal or not; *k* = 1: normal elderly; *k* = 2: PD. In addition, fifteen samples of each class were used as teacher vectors for learning. The discrimination determination threshold *E_d_* was set as 0.4 by trial and error from the discrimination results. Furthermore, to verify the validity of the proposed method for classification, we conducted discrimination experiments using a single LLGMN, a single multilayer perceptron neural network (MLPNN) and 12 MLPNNs combined using the proposed entropy combinator. Generally, the MLPNN has been widely utilized for the pattern discrimination problem. Since the MLP requires sufficient learning and discrimination abilities, we determined its structure based on [10], where the number of layers is 4 and the number of neurons in the hidden layers is 10. Here, the back-propagation method is used as the learning algorithm for the MLPNNs.

### 3.2. Results

**Figure 7.**
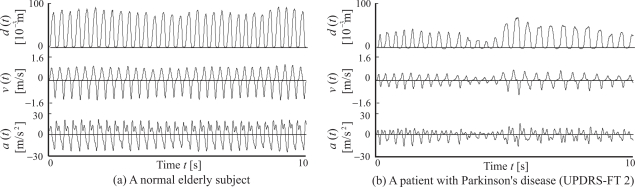
Measured results of finger tapping movements. [12]

**Figure 8.**
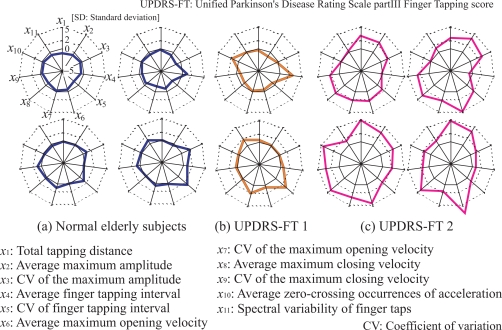
Examples of radar chart representation of the results from the evaluated indices. [12]

**Figure 9.**
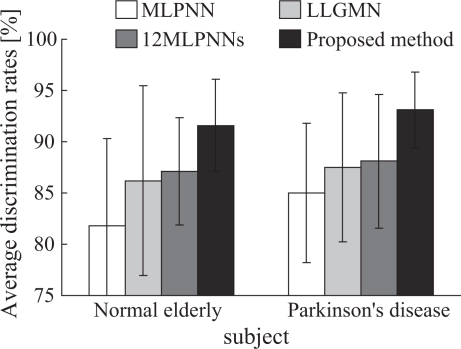
Discrimination rates of finger tapping movements.

**Figure 10.**
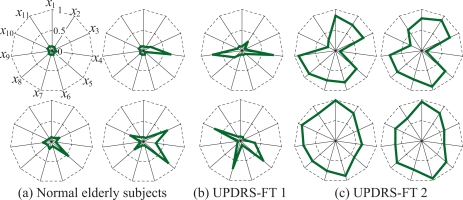
Posteriori probabilities of Parkinson’s disease in each index

Examples of the finger tapping movements of a normal elderly subject (a) and a PD patient (UPDRSFT 2: UPDRS part III Finger Tapping score 2) (b) are shown in Figure 7. The figure plots the measured fingertip distance *d*(*t*), velocity *v*(*t*) and acceleration *a*(*t*), and shows the results of the measured data during the period from 0 to 10 s. Radar chart representation of the results of the indices is shown in Figure 8; (a) to (c) illustrate the charts for normal elderly subjects, PD patients with UPDRS-FT 1 and those with UPDRS-FT 2 respectively. The solid lines describe the average value of each index in the group of normal elderly subjects, and the dotted lines show double and quintuple the standard deviation (2SD, 5SD). The classification results of the finger tapping movements for all subjects are outlined in Figure 9. This shows the mean values and standard deviations of the discrimination rates for 50 kinds of training set and for the test set, where the initial weight coefficients were changed randomly 10 times in each trial. The average discrimination rates of the normal elderly subjects using a single MLPNN, a single LLGMN, 12 MLPNNs and the proposed method were 81.8±8.50%, 86.2±9.24%, 87.1±5.21%, and 91.6 ± 4.51%, and those of the PD patients were 85.0±6.81 %, 87.5±7.25 %, 88.1±6.53 % and 93.1±3.69 %, respectively. The details of the classification results by a single LLGMN and proposed method are explained in Table 1, which shows examples of the results for movements in each subject group. Further, each LLGMN’s output *y*_2_(***x_c_***) (*c* = 1, 2, . . ., 11) (Equation 11), which represents the *a posteriori* probability for PD patients, for all subjects is illustrated in Figure 10. The subjects shown in this figure are the same as those in Figure 8.

### 3.3. Discussion

The experimental results indicate that the finger tapping movements of PD patients and normal elderly subjects have different rhythms and scales. PD patients show a larger variation in tapping rhythm and a smaller scale than normal elderly subjects (Figure 7). Plotting radar charts showing the indices of movements computed and standardized using the data obtained from the normal elderly subjects revealed that data from the normal elderly subjects lie near the average, while those from the PD patients become larger according to the severity of their disease. These results lead us to the conclusion that the radar chart representation can comprehensibly present evaluation results and features of movement.

The results of discrimination demonstrated that the patients could be classified correctly (average rate: 93.1 ± 3.69%) in terms of their impairment status using 12 LLGMNs with a degree of accuracy about 5% higher than the results obtained using a single LLGMN; this outcome shows a higher classification rate than those of other classification methods. Since some elderly subjects were misclassified as PD patients, the average discrimination rate of normal elderly subjects was 91.6 ± 4.51%, which is lower than that of PD patients.

On the other hand, the ratio of discrimination suspension with the proposed method is lower than that of a single LLGMN, and PD patients’ movements can be discriminated more accurately than with a single LLGMN (Table 1). In particular, the discrimination rates for PD patients to normal elderly subjects (i.e., the misclassification rates for PD patients) could be reduced from 15% to 3%, although the average rates for PD patients were improved by only 5 % (Figure 9). These results indicate the effectiveness of the proposed system for possible use in screening tests for patients with PD. Moreover, representing the *a posteriori* probabilities as radar charts confirmed that the values for PD patients become large, and such charts enable quantitative evaluation and description of subjects’ motility function. These results indicate that the proposed method is capable of detecting the disease and supporting PD diagnosis. Here, since the complexity of the system is high compared to the use of a single LLGMN as a classifier, the computational time taken for the learning of all LLGMNs is higher than when a single LLGMN is used. However, in the case of discrimination, it should be noted that the classification of one sample data can be finished in a few milliseconds.

## 4. Conclusion

This paper proposes a diagnosis support system that can quantitatively evaluate motor function for finger tapping movements. The system involves the computation of eleven evaluation indices measured from finger movements and discrimination of the subject’s motor ability.

The results obtained from the experiments using the prototype developed are summarized as follows:

- The proposed system is capable of comprehensibly presenting evaluation results for doctors through visual radar-chart representation of the evaluated results and feature quantities.
- The finger tapping movements of Parkinson’s disease (PD) patients were discriminated with high accuracy (93.1 ± 3.69%), demonstrating that the proposed system is effective in supporting diagnosis using finger movements.
- PD patients’ movements can be discriminated with the proposed method more accurately than with a single probabilistic neural network; this indicates that the proposed system is suitable for use in screening tests for patients with PD.

This paper mainly looks at the difference between the movements of normal elderly subjects and those of PD patients. However, the classification results cannot be presented as showing the relationships between the severity of PD and these movements. In future research, it is therefore necessary to investigate the validity of the proposed method for evaluation and discrimination of the severity of PD by sensing movements. In addition, the effects of the combination rules for the *a posteriori* probabilities output from each LLGMN should be confirmed through comparison with various previous methods [9] such as the median and max rules. To realize a simpler method, it is necessary to discuss a novel PNN that can provide high discrimination performance for motor function evaluation by integrating the multiple-LLGMN structure. We also plan to utilize approximate entropy for the proposed method and to investigate its effectiveness in finger tapping evaluation.

## Figures and Tables

**Table 1.** Examples of the details of classification results with each method.

(a) Single LLGMN				

		Ratio of disc. results		
		NE	PD	Sus.
Subject group	NE	0.719	0.125	0.156
	PD	0.152	0.636	0.212

(b) Proposed method				

		Ratio of disc. results		
		NE	PD	Sus.
Subject group	NE	0.906	0.0625	0.0313
	PD	0.0303	0.909	0.0606

NE: Normal elderly PD: Parkinson's disease Sus. : suspended
